# The onset of posterior reversible encephalopathy syndrome (PRES) and reversible cerebral vasoconstriction syndrome (RCVS) with the use of decongestant agents: a European pharmacovigilance analysis

**DOI:** 10.1007/s43440-026-00877-z

**Published:** 2026-07-10

**Authors:** Antonietta Anatriello, Andrea Cantone, Ciro Pentella, Maria Giovanna Vastarella, Annamaria Mascolo, Annalisa Capuano, Concetta Rafaniello

**Affiliations:** 1 Campania Regional Centre for Pharmacovigilance and Pharmacoepidemiology, Napoli, Italy; 2 Department of Experimental Medicine, Section of Pharmacology “L. Donatelli”, University of Campania “Luigi Vanvitelli”, Napoli, Italy; 3 Department of Women, Child and General and Special Surgery, University of Campania “Luigi Vanvitelli”, Naples, 80138 Italy; 4 Department of Life Sciences, Health, and Health Professions, Link Campus University, Rome, Italy; 5 Campania Regional Centre for Pharmacovigilance and Pharmacoepidemiology, Department of Experimental Medicine, Section of Pharmacology “L. Donatelli”, University of Campania “Luigi Vanvitelli”, Via Costantinopoli 16, Naples, 80138 Italy

**Keywords:** Decongestants, Posterior reversible encephalopathy syndrome, Reversible cerebral vasoconstriction syndrome, Safety, Pharmacovigilance

## Abstract

**Background:**

On December 1st, 2023, the Pharmacovigilance Risk Assessment Committee (PRAC) published a safety warning against using pseudoephedrine in patients with severe or uncontrolled hypertension and severe acute or chronic kidney disease for the risk of developing posterior reversible encephalopathy syndrome (PRES) or reversible cerebral vasoconstriction syndrome (RCVS). Based on this consideration and the availability of other decongestants with the same mechanism of action as pseudoephedrine in the European market, we decided to conduct a safety study to evaluate cases of RCVS or PRES reported with other decongestants.

**Methods:**

A European, retrospective, post-marketing surveillance study was conducted to describe individual case safety reports (ICSRs) of RCVS and PRES reported with decongestants and to compare the likelihood of reporting these events among decongestants. Data were retrieved from the EudraVigilance database from January 1st, 2001, to December 31st, 2025.

**Results:**

A total of 65 ICSRs reported PRES or RCVS with decongestants, most of which referred to female patients aged 18–64 years. A disproportionate reporting of RCVS was found for xylometazoline (reporting odds ratio, ROR: 3.39; 95% confidence interval, CI: 1.94–5.93) and phenylephrine (ROR: 2.00; 95% CI: 1.11–3.61), and a disproportionate reporting of PRES for ephedrine (ROR: 6.44; 95% CI: 1.80–23.10) and phenylephrine (ROR: 3.47; 95% CI: 1.22–9.88) compared to all other decongestants.

**Conclusion:**

Our results suggest that all decongestants should be used with caution, and attention should be paid to signs and symptoms of PRES and RCVS when taking these medicines.

**Supplementary Information:**

The online version contains supplementary material available at 10.1007/s43440-026-00877-z.

## Introduction

The Pharmacovigilance Risk Assessment Committee (PRAC) of the European Medicines Agency’s (EMA) started, at the request of the French Medicines Agency (ANSM), a safety review of all pharmaceutical formulations containing the decongestant pseudoephedrine to assess if there was a potential causal association between the aforementioned products and posterior reversible encephalopathy syndrome (PRES) or reversible cerebral vasoconstriction syndrome (RCVS) [1]. RCVS is a cerebrovascular disease clinically presenting with recurrent episodes of sudden-onset thunderclap headaches, with or without other neurological deficits [2]. Common risk factors for RCVS are postpartum status, eclampsia, pre-eclampsia, immunosuppression, and the use of vasoactive substances [3, 4]. The definite incidence of RCVS is still unknown, probably because it is under- and/or misdiagnosed; indeed, RCVS frequently overlaps with PRES, whose clinical manifestations are headache, visual alterations, and seizure. The key risk factors for the development of PRES are hypertension and associated conditions; however, there are also normo- or hypotensive patients who can develop PRES [5]. Other risk factors associated with PRES are preeclampsia/eclampsia, renal dysfunction, cytotoxic drugs, systemic infections, and autoimmune diseases [5].

On December 1st, 2023, the PRAC concluded that pseudoephedrine is associated with risks of PRES and RCVS and recommended a safety warning of not using pseudoephedrine in patients with severe or uncontrolled hypertension and severe acute or chronic kidney disease [6].

Pseudoephedrine, like other decongestants (such as naphazoline, ephedrine, oxymetazoline, and xylometazoline) available on the market as nasal or oral formulations, alone or in association with non-steroidal anti-inflammatory drugs (e.g. ibuprofen or paracetamol) or antihistamines (triprolidine), exerts its effect through the activation of postjunctional alpha (α)-adrenergic receptors mainly expressed on the capillaries of the nasal mucosa. The activation of these receptors occurs by either a direct binding to the receptor site or by potentiating the release of norepinephrine. These effects can induce vasoconstriction and reduce nasal congestion [7], making these medicines widely used for the common cold and vasomotor rhinitis, both in adults and adolescents [8]. Moreover, as these medicines are available over the counter (OTC), their use has become widespread and often uncontrolled over the years, driven by the misconception that they are inherently safe simply because they can be obtained without a prescription [9]. On the contrary, many OTC medicines, including decongestants, have shown the potential for abuse, misuse, and adverse effects [10], highlighting the need for safety monitoring.

Based on this consideration, the common mechanism of action of all decongestants and the association between RCVS/PRES and pseudoephedrine, we decided to conduct a study to evaluate cases of RCVS or PRES reported with other decongestants available on the European market.

## Materials and methods

### Study design

A European, retrospective, post-marketing, surveillance study aiming to (1) describe cases of RCVS and PRES reported with decongestants and (2) compare the likelihood of reporting these events among decongestants.

### Data source

All available information on cases of RCVS and PRES was retrieved from the EudraVigilance (EV) database from January 1st, 2001, to December 31st, 2025. EV is a database of suspected adverse drug reaction (ADR) reports accessible online through the official website (https://www.adrreports.eu/en/data_source.html) and managed by EMA. EV collects individual case safety reports (ICSRs) related to medicines or vaccines authorized or undergoing clinical trials in the European Economic Area (EEA). We used a level of access dedicated to the stakeholder group II (SGII: Healthcare Professionals and the Public) based on the revised EV access policy. Through this website, it is possible to view all ICSRs as aggregated data or line listings based on restricted data elements that include information on the geographic origin, reporter, age, sex, adverse reaction, suspected and concomitant drugs [11]. According to the International Council for Harmonisation of Technical Requirements for Pharmaceuticals for Human Use (ICH) guideline E2B (R3) on electronic transmission of ICSRs, a minimum of one suspect medication needs to be provided for each valid ICSR. The role reported for a drug in an ICSR is implied by the primary reporter (e.g., the original source of the information). Suspected medications are those medicinal products taken by the patient and suspected by the reporter to have contributed to an ADR. Concomitant medications are, instead, those medicines taken by the patient at the time the ADR is observed and not considered related to it [12].

### Selection of ICSRs with line listing

All ICSRs of interest were retrieved by using the line-listing function of EV. An ICSR was defined as of interest if reporting a decongestant among suspected medicines, and RCVS or PRES among ADRs. To identify the events of interest among ICSRs, the preferred terms (PTs) of Medical Dictionary for Regulatory Activities (MedDRA^®^) “RCVS” or “PRES” were used.

Decongestants were identified through the Anatomical Therapeutic Chemical (ATC) classification “R01A - Decongestants and other nasal preparations for topical use” and “R01B - Nasal decongestants for systemic use”. The full list of decongestants searched in EV is shown in supplementary Table 1. The search for RCVS and PRES was performed for each decongestant, then the retrieved single decongestant datasets were merged with duplicate removal.

### Descriptive analyses

Information on patient characteristics (age and sex), adverse event (in terms of outcome and seriousness), primary source qualification (healthcare- and non-healthcare professionals – HCP or non-HCP), primary source country for regulatory purposes (EEA or non-EEA), number of suspected drugs, and number of concomitant drugs were provided for all ICSRs and separately for each identified decongestant. The seriousness and the outcome of an ADR were defined according to the ICH E2D guidelines. Specifically, a case was defined as serious when the suspected ADR (1) resulted in death, (2) was life-threatening, (3) required hospitalization or prolongation of an existing hospitalization, (4) resulted in persistent or significant disability/incapacity, (5) was a congenital anomaly/birth defect, or (6) resulted in some other medically important condition. The outcome of ADRs was classified into recovered/resolved, recovering/resolving, recovered/resolved with sequelae, not recovered/not resolved, fatal, and unknown. The therapy duration of decongestants, as median days and interquartile range (IQR), was also evaluated (when available) to identify cases of inappropriate use.

ICSRs were also evaluated with a case-by-case approach to identify risk factors for RCVS and PRES, considering information on concomitant and suspected drugs (other than decongestant ones). Risk factors also considered were postpartum status, eclampsia/pre-eclampsia, immunosuppression, renal dysfunction, cytotoxic drugs, systemic infections, autoimmune diseases, and hypotension.

### Disproportionality analyses

To compare the likelihood of reporting PRES and RCVS for a decongestant, the reporting odds ratio (ROR) and its 95% confidence interval (95% CI) were calculated in accordance with READUS-PV guideline and EMA guidance [13, 14]. The disproportionality analysis relies on a 2 × 2 contingency table approach to identify potential safety signals, and it was performed on drug-event couples [15]. We computed the ROR to compare the proportion of RCVS or PRES associated with the decongestant of interest (e.g., oxymetazoline) against the proportion of those events linked to all other decongestants in our dataset (comparator group). The comparator group was chosen considering our level of access to EV and to reduce confounding by indication [15]. The ROR was computed as follows: $\:ROR=\frac{a/b}{c/d}$.

**Table:** 

	RCVS or PRES	Other adverse events	Total
Decongestant of interest	a	b	a + b
Comparator	c	d	c + d
Total	a + c	b + d	a + b+c + d

This computation was applied only for decongestant-event (RCVS or PRES) couples reported at least three times in the database. A threshold of less than 0.05 was chosen for statistical significance. Data management and data analyses were performed with R version 4.3.1 (R Foundation for Statistical Computing, Vienna, Austria).

**Fig. 1:**
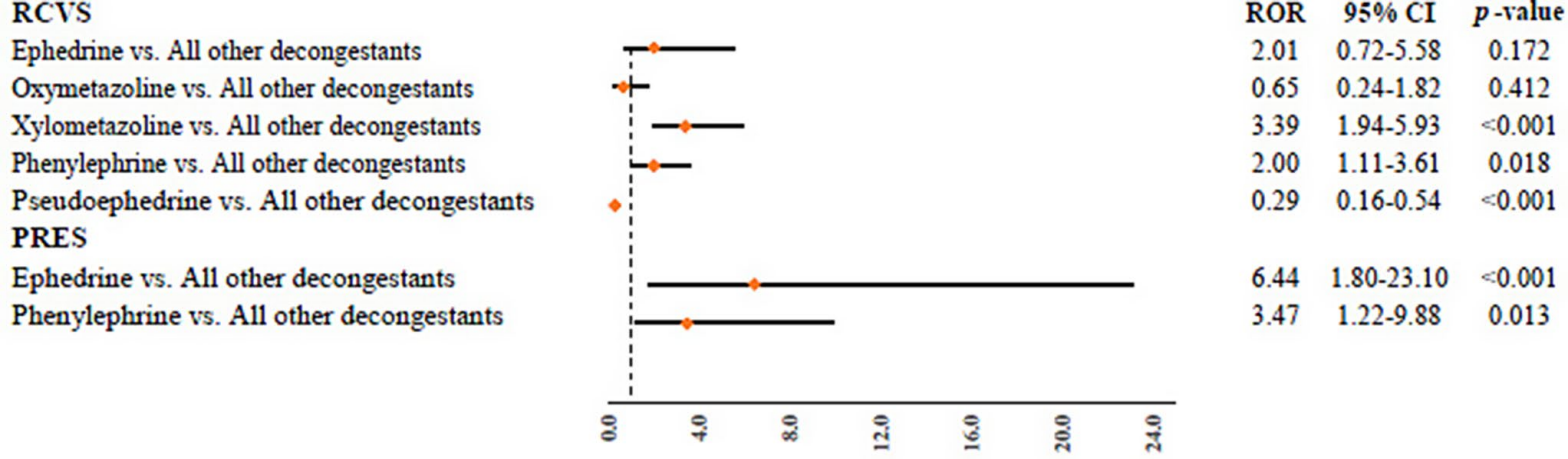
RORs and their 95% CI of RCVS and PRES for a decongestant compared to all other decongestants. The ROR computation was performed only for decongestant-event couples reported at least three times in the database. 95% CI: confidence interval, PRES: posterior reversible encephalopathy syndrome, RCVS: reversible cerebral vasoconstriction syndrome, ROR: reporting odds ratio

### Compliance with ethical standards

Safety data derived from the spontaneous reporting system are anonymous and in compliance with ethical standards; therefore, no further ethical measures were required.

## Results

During the study period, we found a total of 65 (0.4%) of 14,628 ICSRs reporting PRES or RCVS with a decongestant. Specifically, most ICSRs reported one decongestant formulation among suspected medicines (*n* = 58; 89.2%) with the following decongestants identified: phenylephrine (*n* = 18; 27.6%), xylometazoline (*n* = 15; 23.1%), pseudoephedrine (*n* = 10; 15.4%), oxymetazoline (*n* = 5; 7.7%), ephedrine (*n* = 5; 7.7%), fixed-dose formulation of pseudoephedrine/ ibuprofen (*n* = 3; 4.6%), and fixed-dose formulation of pseudoephedrine/ paracetamol/ triprolidine (*n* = 2; 3.1%). The remaining ICSRs (*n* = 7; 10.7%), reporting more than one decongestant formulation among suspected medicines, included three cases with two suspected decongestants (phenylephrine and pseudoephedrine; or phenylephrine and ephedrine; or pseudoephedrine and xylometazoline), and four cases with three suspected decongestants (pseudoephedrine, phenylephrine, and xylometazoline). The route of administration was unknown (*n* = 25) in most ICSRs, followed by systemic (*n* = 22) and nasal administration (*n* = 18).

As reported in Table 1, most ICSRs referred to patients aged 18–64 years (95.4%) and female (84.6%). The primary source qualification was represented by HCPs (92.3%), while the primary source country for regulatory purposes was the non-EEA (70.8%). In 40% of ICSRs, decongestants were the only suspected drug reported. Most ICSRs (55.4%) did not report any concomitant medications. Overall, 13 ICSRs were related to PRES, 51 to RCVS, and one ICSR reported both. All ICSRs were classified as serious. Seriousness and outcome criteria for RCVS and PRES are reported in Table 2.

**Table 1:** Distribution of ICSRs related to PRES and RCVS in patients treated with nasal decongestants by age group, sex, primary source (reporter), primary source country for regulatory purposes, presence of other suspected or concomitant drugs, and events of RCVS and PRES, and reported in EudraVigilance from 2001 to December 31st, 2025

	EPHE (*N* = 5)	OXY (*N* = 5)	PHEN (*N* = 18)	PHEN and EPHE* (*N* = 1)	PHEN and PSEUDO* (*N* = 1)	PSEUDO-IBU** (*N* = 3)	PSEUDO-PARA-TRIP** (*N* = 2)	PSEUDO (*N* = 10)	XYLO (*N* = 15)	XYLO, PHEN, and PSEUDO* (*N* = 4)	XYLO, and PSEUDO* (*N* = 1)	OVERALL (*N* = 65)
**Age Group**												
18–64 Years	5 (100%)	5 (100%)	16 (88.9%)	1 (100%)	1 (100%)	3 (100%)	2 (100%)	10 (100%)	15 (100%)	3 (75.0%)	1 (100%)	62 (95.4%)
65–85 Years	0 (0%)	0 (0%)	1 (5.6%)	0 (0%)	0 (0%)	0 (0%)	0 (0%)	0 (0%)	0 (0%)	0 (0%)	0 (0%)	1 (1.5%)
> 85 Years	0 (0%)	0 (0%)	1 (5.6%)	0 (0%)	0 (0%)	0 (0%)	0 (0%)	0 (0%)	0 (0%)	0 (0%)	0 (0%)	1 (1.5%)
Not Specified	0 (0%)	0 (0%)	0 (0%)	0 (0%)	0 (0%)	0 (0%)	0 (0%)	0 (0%)	0 (0%)	1 (25.0%)	0 (0%)	1 (1.5%)
**Sex**												
Female	4 (80.0%)	4 (80.0%)	15 (83.3%)	1 (100%)	1 (100%)	3 (100%)	1 (50.0%)	9 (90.0%)	12 (80.0%)	4 (100%)	1 (100%)	55 (84.6%)
Male	1 (20.0%)	1 (20.0%)	3 (16.7%)	0 (0%)	0 (0%)	0 (0%)	1 (50.0%)	1 (10.0%)	3 (20.0%)	0 (0%)	0 (0%)	10 (15.4%)
**Reporter**												
HCP	5 (100%)	5 (100%)	17 (94.4%)	1 (100%)	1 (100%)	3 (100%)	2 (100%)	9 (90.0%)	15 (100%)	2 (50.0%)	0 (0%)	60 (92.3%)
Non-HCP	0 (0%)	0 (0%)	1 (5.6%)	0 (0%)	0 (0%)	0 (0%)	0 (0%)	1 (10.0%)	0 (0%)	2 (50.0%)	1 (100%)	5 (7.7%)
**Country**												
EEA	4 (80.0%)	2 (40.0%)	3 (16.7%)	0 (0%)	0 (0%)	3 (100%)	2 (100%)	3 (30.0%)	1 (6.7%)	1 (25.0%)	0 (0%)	19 (29.2%)
Non-EEA	1 (20.0%)	3 (60.0%)	15 (83.3%)	1 (100%)	1 (100%)	0 (0%)	0 (0%)	7 (70.0%)	14 (93.3%)	3 (75.0%)	1 (100%)	46 (70.8%)
**Concomitants**												
0	3 (60.0%)	2 (40.0%)	11 (61.1%)	0 (0%)	0 (0%)	2 (66.7%)	2 (100%)	8 (80.0%)	5 (33.3%)	3 (75.0%)	0 (0%)	36 (55.4%)
1	2 (40.0%)	0 (0%)	3 (16.7%)	0 (0%)	1 (100%)	0 (0%)	0 (0%)	0 (0%)	0 (0%)	1 (25.0%)	1 (100%)	8 (12.3%)
2	0 (0%)	0 (0%)	2 (11.1%)	0 (0%)	0 (0%)	0 (0%)	0 (0%)	1 (10.0%)	6 (40.0%)	0 (0%)	0 (0%)	9 (13.8%)
3	0 (0%)	0 (0%)	1 (5.6%)	1 (100%)	0 (0%)	0 (0%)	0 (0%)	0 (0%)	1 (6.7%)	0 (0%)	0 (0%)	3 (4.6%)
4	0 (0%)	1 (20.0%)	1 (5.6%)	0 (0%)	0 (0%)	0 (0%)	0 (0%)	1 (10.0%)	0 (0%)	0 (0%)	0 (0%)	3 (4.6%)
≥ 5	0 (0%)	2 (40.0%)	0 (0%)	0 (0%)	0 (0%)	1 (33.3%)	0 (0%)	0 (0%)	3 (20.0%)	0 (0%)	0 (0%)	6 (9.2%)
**Suspects**												
1	1 (20.0%)	0 (0%)	12 (66.7%)	0 (0%)	0 (0%)	1 (33.3%)	1 (50.0%)	8 (80.0%)	2 (13.3%)	1 (25.0%)	0 (0%)	26 (40.0%)
2	2 (40.0%)	1 (20.0%)	3 (16.7%)	0 (0%)	0 (0%)	2 (66.7%)	0 (0%)	2 (20.0%)	6 (40.0%)	1 (25.0%)	1 (100%)	18 (27.7%)
3	2 (40.0%)	3 (60.0%)	2 (11.1%)	0 (0%)	0 (0%)	0 (0%)	1 (50.0%)	0 (0%)	1 (6.7%)	1 (25.0%)	0 (0%)	10 (15.4%)
4	0 (0%)	1 (20.0%)	1 (5.6%)	0 (0%)	0 (0%)	0 (0%)	0 (0%)	0 (0%)	4 (26.7%)	0 (0%)	0 (0%)	6 (9.2%)
≥ 5	0 (0%)	0 (0%)	0 (0%)	1 (100%)	1 (100%)	0 (0%)	0 (0%)	0 (0%)	2 (13.3%)	1 (25.0%)	0 (0%)	5 (7.7%)
**RCVS**	3^#^ (50.0%)	4 (80.0%)	12 (66.7%)	1 (100.0%)	1 (100.0%)	2 (66.7%)	2 (100.0%)	8 (80.0%)	15 (100.0%)	4 (100.0%)	1 (100.0%)	53 (80.3%)
**PRES**	3^#^ (50.0%)	1 (20.0%)	6 (33.3%)	0 (0%)	0 (0%)	1 (33.3%)	0 (0%)	2 (20.0%)	0 (0%)	0 (0%)	0 (0%)	13 (19.7%)

All values were presented as numbers (N) and percentages (%) EEA: European economic area, EPHE: ephedrine, HCP: healthcare professional, IBU: ibuprofen, ICSRs: individual case safety reports, OXY: oxymetazoline, PARA: paracetamol, PHEN: phenylephrine, PRES: posterior reversible encephalopathy syndrome, PSEUDO; pseudoephedrine, RCVS: reversible cerebral vasoconstriction syndrome, TRIP: triprolidine, XYLO: xylometazoline

*ICSRs reporting different formulations containing a single decongestant (more than 1 decongestant reported as suspected drug)

**ICSRs reporting a single formulation containing more active ingredients

^#^ 1 ICSRS reported both RCVS and PRES

**Table 2:** Seriousness and outcome criteria for PRES and RCVS distributed for decongestants and reported in Eudravigilance from 2001 to December 31st, 2025

	EPHE (*N* = 5)	OXY (*N* = 5)	PHEN (*N* = 18)	PHEN and EPHE* (*N* = 1)	PHEN and PSEUDO* (*N* = 1)	PSEUDO-IBU** (*N* = 3)	PSEUDO-PARA-TRIP** (*N* = 2)	PSEUDO (*N* = 10)	XYLO (*N* = 15)	XYLO, PHEN, and PSEUDO* (*N* = 4)	XYLO, and PSEUDO* (*N* = 1)	OVERALL (*N* = 65)
**Seriousness**												
Caused/Prolonged Hospitalisation	2 (40.0%)	4 (80.0%)	9 (50.0%)	1 (100%)	1 (100%)	2 (66.7%)	2 (100%)	8 (80.0%)	12 (80.0%)	1 (25.0%)	0 (0%)	42 (64.6%)
Other Medically Important Condition	3 (60.0%)	1 (20.0%)	5 (27.8%)	0 (0%)	0 (0%)	0 (0%)	0 (0%)	2 (20.0%)	0 (0%)	1 (25.0%)	0 (0%)	12 (18.5%)
Life-Threatening	0 (0%)	0 (0%)	4 (22.2%)	0 (0%)	0 (0%)	0 (0%)	0 (0%)	0 (0%)	3 (20.0%)	2 (50.0%)	1 (100%)	10 (15.4%)
Disabling	0 (0%)	0 (0%)	0 (0%)	0 (0%)	0 (0%)	1 (33.3%)	0 (0%)	0 (0%)	0 (0%)	0 (0%)	0 (0%)	1 (1.5%)
**Outcome**												
Recovered/Resolved	3 (60.0%)	4 (80.0%)	10 (55.6%)	1 (100%)	0 (0%)	0 (0%)	1 (50.0%)	7 (70.0%)	10 (66.7%)	1 (25.0%)	0 (0%)	37 (56.9%)
Recovered/Resolved With Sequelae	1 (20.0%)	0 (0%)	0 (0%)	0 (0%)	1 (100%)	1 (33.3%)	1 (50.0%)	0 (0%)	0 (0%)	0 (0%)	0 (0%)	4 (6.2%)
Recovering/Resolving	1 (20.0%)	0 (0%)	3 (16.7%)	0 (0%)	0 (0%)	1 (33.3%)	0 (0%)	0 (0%)	0 (0%)	1 (25.0%)	0 (0%)	6 (9.2%)
Unknown	0 (0%)	1 (20.0%)	5 (27.8%)	0 (0%)	0 (0%)	1 (33.3%)	0 (0%)	3 (30.0%)	3 (20.0%)	2 (50.0%)	1 (100%)	16 (24.6%)
Not Recovered/Not Resolved	0 (0%)	0 (0%)	0 (0%)	0 (0%)	0 (0%)	0 (0%)	0 (0%)	0 (0%)	2 (13.3%)	0 (0%)	0 (0%)	2 (3.1%)

All values were presented as numbers (N) and percentages (%). EPHE: ephedrine, IBU: ibuprofen, ICSRs: individual case safety reports, OXY: oxymetazoline, PARA: paracetamol, PHEN: phenylephrine, PRES: posterior reversible encephalopathy syndrome, PSEUDO; pseudoephedrine, RCVS: reversible cerebral vasoconstriction syndrome, TRIP: triprolidine, XYLO: xylometazoline

* ICSRs reporting different formulations containing a single decongestant (more than 1 decongestant reported as suspected drug)

**ICSRs reporting a single formulation containing more active ingredients

Since one ICSR could describe more than one suspected adverse reaction, a total of 286 adverse events were identified. The most reported events other than PRES and RCVS were headache (5.9%), subarachnoid hemorrhage (4.2%), thunderclap headache (3.8%), hypertension (2.8%), cerebral hemorrhage (2.8%), cerebral vasoconstriction (2.1%), aphasia (1.4%), and vomiting (1.7%). All events are listed in supplementary Table 2. A total of 76 events were reported with xylometazoline, 72 with phenylephrine, 37 with pseudoephedrine, 27 with ephedrine, 21 with oxymetazoline, 16 with both phenylephrine and pseudoephedrine, 12 events with both ephedrine and phenylephrine, 12 with pseudoephedrine/ ibuprofen, six with both xylometazoline, phenylephrine, and pseudoephedrine, five with pseudoephedrine/ paracetamol/ triprolidine and two with both pseudoephedrine and xylometazoline (Supplementary Table 2).

Information on the duration of decongestant therapy was available for 12 out of 65 ICSRs (18.5%). Specifically, the following data about therapy duration were reported: phenylephrine and pseudoephedrine (both reported as suspected) used for 30 days; phenylephrine, pseudoephedrine, and xylometazoline for 37 days; fixed-dose combination of pseudoephedrine/ paracetamol/ triprolidine for seven days; phenylephrine for two days; oxymetazoline and pseudoephedrine for one day; fixed-dose combination of pseudoephedrine/ ibuprofen for 18 h. Three ICSRs reported oxymetazoline with a duration of use of six months; finally, one ICSR reported the duration of six years for xylometazoline. Moreover, regarding the last ICSR, it is important to highlight that the PT “Intentional product misuse” was reported among adverse events (Supplementary Table 2). Analyzing all the available data about duration of use, the median duration was 30 days (IQR: 182 − 1.75).

### Reporting probability of PRES and RCVS among identified decongestants

RCVS was reported at least three times for xylometazoline (*n* = 20), phenylephrine (*n* = 17), pseudoephedrine (*n* = 14), oxymetazoline (*n* = 4), and ephedrine (*n* = 4). PRES was reported at least three times only for phenylephrine (*n* = 7) and ephedrine (*n* = 3). A disproportionate reporting of RCVS was observed for xylometazoline (ROR: 3.39; 95% CI: 1.94–5.93) and phenylephrine (ROR: 2.00; 95% CI: 1.11–3.61), and a disproportionate reporting of PRES for ephedrine (ROR: 6.44; 95% CI: 1.80–23.10) and phenylephrine (ROR: 3.47; 95% CI: 1.22–9.88). All results of the disproportionality analyses are shown in Fig. 1.

### Concomitant conditions

In the evaluation of risk factors, we found 31 out of 65 ICSRs reporting suspected/concomitant drugs or other conditions that can predispose to PRES and RCVS for a total of 39 contributing risk factors. Specifically, we found cardiovascular medicines among suspected drugs, including verapamil (*n* = 2), and nimodipine (*n* = 1). Among cardiovascular concomitant drugs, verapamil (*n* = 1), a combination of metoprolol, nimodipine, and valsartan (*n* = 1), a combination of metoprolol and valsartan (*n* = 1), losartan (*n* = 1), nimodipine (*n* = 2), and a combination of spironolactone, hydralazine, isosorbide dinitrate, and nitroprusside (*n* = 1) were identified. Two ICSRs involved pregnant women, suggesting a potential condition of gestational hypertension as a contributing risk factor. Three ICSRs reported a concomitant infection, three ICSRs also reported hypotension, and one ICSR reported chronic kidney disease. Moreover, 20 ICSRs also reported antidepressant drugs among suspected or concomitant drugs, which were duloxetine, sertraline, escitalopram, and venlafaxine.

## Discussion

In this study, we investigated the safety profile of decongestants, considering specifically the reporting frequency of PRES and RCVS in the European database, EV. We found a low number of ICSRs reporting PRES and RCVS with decongestants as suspected drugs, probably associated with the rarity of such events [3]. Our main results revealed a higher number of cases with phenylephrine and xylometazoline, and a disproportionate reporting of RCVS for these decongestants compared to all the others. This higher reporting frequency may be explained by the preferential topical use of xylometazoline and phenylephrine, which can accelerate drug absorption and the maximum plasma concentration, causing rapid, localized, central vasoconstriction. A meta-analysis demonstrated, indeed, a correlation between high doses of decongestant and an increase in systolic blood pressure [16]. Hence, suggesting that an extension of half-life, immediate-release formulations, or accidental overdoses may cause intracranial vasoconstriction and RCVS/PRES. Despite differences in the pharmacokinetics between oral and nasal decongestants [17], we found too few ICSRs to evaluate and compare different formulations. Moreover, we found a disproportionate reporting of PRES for ephedrine and phenylephrine compared to all other decongestants, which are both potent sympathomimetic agents that can cause acute hypertension and endothelial dysfunction, leading to PRES [18], with phenylephrine also primarily acting on α-1 adrenergic receptors [19]. Moreover, we observed disproportionate reporting for RCVS with all other decongestants compared to pseudoephedrine, highlighting that all decongestants can be potentially associated with an even higher risk. These results suggest a potential risk of RCVS and PRES with all decongestants and the need to further evaluate this risk through specific epidemiological studies. In the literature, indeed, no study has compared this risk among different decongestants. Only case reports have been published with systemic or topical use. A case report was published showing the onset of PRES after the use of oral paracetamol and pseudoephedrine for a runny nose, sore throat, and headache [20]. Similarly, another case report hypothesized the contribution of oral pseudoephedrine in determining hypertension, endothelial dysfunction, and vasogenic edema related to PRES [21]. Cases of overdose with the onset of hypertension, coronary vasospasm, acute myocardial infarction, or acute ischemic colitis have also been reported in the literature [22], as well as cases of pseudoephedrine-induced RCVS [23, 24].

A case report referred to the intravenous administration of phenylephrine during surgery for the treatment of general anesthesia-induced hypotension [25]. Another case series described the association of phenylephrine with both typical and atypical presentations of PRES [26]. An important aspect of ephedrine and phenylephrine is their use for cardiovascular indications, making our analysis non-exempt from confounding by indication for these two decongestants. Moreover, a case report showed the onset of recurrent thunderclap headaches after treatment with duloxetine and xylometazoline in a 52-year-old woman. In this case, rhinitis medicamentosa, a medicine-induced rhinitis due to the overuse of topical α-adrenergic receptor agonists (such as nasal decongestants) and characterized by rebound tachyphylaxis and nasal congestion, was also diagnosed. This disease can predispose patients to a vicious circle of refractory nasal congestion, which could indirectly predispose to RCVS [27]. Another case report described the onset of ischemic stroke after the chronic misuse of nasal spray xylometazoline [28]. These case reports suggest a role of the inappropriate use of nasal decongestants in the onset of PRES and RCVS, which was also observed on the therapy duration in our cases, and it was far greater than the maximum recommended duration of 7 days at a time.

According to our results, PRES and RCVS occurred most frequently in female patients; indeed, regarding PRES, even if there is no clear male/female prevalence, data suggested that women tend to be at higher risk [29]. Another study showed the predominance of RCVS in females rather than males [30]. Although evidence explaining this apparent gender difference in terms of RCVS as well as PRES is not available, it is nowadays well recognized that ADR prevalence is higher in women than men; in that regard, several hypotheses have been proposed, such as sexual hormones and physiological differences that could lead to changes in terms of drug safety profile independently from the drug class [31].

The age group most represented was 18–64 years, in line with marketing authorization details recommending the use of oxymetazoline [32] and xylometazoline [33] in patients aged more than 12 years, while the association pseudoephedrine/ibuprofen in patients more than 15 years [34]. The safety and efficacy of phenylephrine in children have not been established, and it is contraindicated in children aged less than 12 years [35].

The HCPs were the main source of reporting, in accordance with other studies [36, 37]. All ICSRs were classified as serious not only for hospitalization, but especially because PRES and RCVS are included in the Important Medical Event (IME) list (version 24.1) of EMA. Moreover, the most common reported events, like cerebral hemorrhage, subarachnoid hemorrhage, hemiparesis, cerebral artery stenosis, and seizure, which are also symptoms of both syndromes, are in the IME list as well. In most ICSRs, PRES and RCVS had a complete resolution, despite an unknown outcome. This result may be explained by the fact that vasoconstriction of cerebral arteries is prolonged in RCVS, but still reversible. Headache is the main symptom, and it can occur intermittently for days or weeks, resolving completely in approximately 90% of patients [38]. Regarding PRES, prognosis is generally favorable, and most patients make a full recovery, though some diagnostic criteria require clinical and radiographic resolution. Complete recovery is reported in 75–90% of cases, and most patients recover within a week, though in some patients, recovery occurs over a longer interval, and neurological sequelae have been reported in 10–20% of patients [39]. Another aspect is the presence of other concomitant conditions that are risk factors for PRES and RCVS. Among our cases, almost half reported a concomitant risk factor. We found the use of antihypertensive drugs, such as verapamil, valsartan, and losartan. Although anti-hypertensive drugs, especially calcium channel blockers, are used for the treatment of PRES and RCVS [40], they can also be indicative of patients suffering from hypertension (a precipitant risk factor) who may require an antihypertensive medication review [41], implying that these medicines may not be protective in a subgroup of patients with resistant hypertension, but further studies must be conducted to deepen this aspect. We found gestational risk factors in two cases. Particularly, in pregnancy, hypertension is a condition related to some disorders, such as preeclampsia, and when it involves neurologic problems, such as seizures, it becomes eclampsia. Both syndromes are risk factors for PRES and RCVS. Indeed, the study of Liman et al. [42] described the clinical features of PRES in patients with preeclampsia/eclampsia. Lihong et al. also described a case report of a 28-year-old woman diagnosed with pre-eclampsia and PRES after magnetic resonance imaging [43]. Additionally, we found antidepressants as other contributing factors. It is well known that antidepressants are vasoactive substances, which have been identified as important risk and precipitant factors for the onset of RCVS [44]. This may be linked to the proinflammatory effects of serotonin, which can lead to vasoconstriction and excessive capillary permeability, compromising the blood–brain barrier function and inducing vasogenic edema [45]. Moreover, the Summary of Product Characteristics of the antidepressant sertraline includes this rare adverse event among the safety information [46]. Finally, it is also important to underline that EV data concerning suspected ADRs are not necessarily related to or caused by the drug. Therefore, further well-designed studies may be needed to specifically investigate the risk of RCVS or PRES with decongestant agents.

### Strengths and limitations

Our study has some strengths and limitations. Among the strengths, we should consider that the spontaneous reporting system can detect ADRs not detectable during the pre-marketing phase, including rare and serious ones, such as RCVS and PRES [47, 48]. Therefore, through the EV database, we have analyzed many ICSRs related to decongestants and the entire European area. Moreover, the identification of RCVS and PRES via MedDRA PTs provided a consistent, objective approach to outcome classification, thereby minimizing information bias (misclassification) and improving reproducibility. Among limitations, we have the poor quality of information listed in each ICSR, such as the route of administration, the number of sprays or squeezes for nasal use, the dose, the therapeutic indication, and information on risk factors (concomitant drugs or diseases) that may be missing. Another major limitation is under-reporting, or rather the failure to submit ADRs to regulatory authorities or manufacturers, which can underestimate the frequency of an ADR. This limitation can be even higher for OTC medicines that are widely available and perceived as safe, with consumers rarely linking them to adverse events or knowing how to report them to a regulatory authority. Another limitation is the rarity of these events (low number of ICSRs), which can make our estimates imprecise with wide confidence intervals. Moreover, codification bias can occur in our study due to the inconsistent translation of clinical narratives into standardized medical terms (RCVS and PRES), leading to an underestimation of cases. This misclassification can undermine signal detection, potentially causing regulators to miss drug risks or wrongly associate medicines with harm. Finally, confounding by indication, comorbidities, and reporting bias in spontaneous-reporting systems may affect disproportionality analyses and signal detection, requesting other targeted epidemiological studies to assess and confirm causality.

## Conclusion

We found that all decongestants may be associated with a higher reporting of RCVS and PRES, highlighting the need for constant and continuous monitoring of patients treated with these medicines, especially for those with pre-existing risk factors. Indeed, almost half of these cases reported contributing factors for RCVS and PRES. Despite the difficulty in evaluating inappropriate use due to the few cases, data published in the literature also suggested it, and considering the severity of these events, more restrictive measures should be adopted in selling OTC decongestants. Being a pharmacovigilance study, we cannot speculate on the mechanisms underlying the onset of RCVS and PRES with decongestants. However, the key message is that attention should be paid to all decongestants, and further evaluation on this topic is required. Patients must be informed of the signs and symptoms of PRES and RCVS, such as thunderclap headache, confusion, changes in vision, difficulty in speaking, weakness, vomiting, and seizures, when taking these medicines, and to alert HCP when they occur.

## Supplementary Information

Below is the link to the electronic supplementary material.

Supplementary Material 1

## Data Availability

The datasets generated during the current study are available at the https://www.adrreports.eu/en/search.html and from the corresponding author upon reasonable request.

## Abbreviations

95% CI: 95% confidence interval
ADR: Adverse drug reaction
ATC: Anatomical Therapeutic Chemical
EEA: European Economic Area
EMA: European Medicines Agency
EV: EudraVigilance
HCP: Healthcare professionals
ICH: International Council for Harmonisation of Technical Requirements for Pharmaceuticals for Human Use
ICSR: Individual case safety report
IME: Important Medical Event
IQR: Interquartile range
MedDRA: Medical Dictionary for Regulatory Activities
OTC: Over the counter
PRAC: Pharmacovigilance Risk Assessment Committee
PRES: Posterior reversible encephalopathy syndrome
PT: Preferred term
RCVS: Reversible cerebral vasoconstriction syndrome
ROR: Reporting odds ratio
