# Supplementary material for: The onset of posterior reversible encephalopathy syndrome (PRES) and reversible cerebral vasoconstriction syndrome (RCVS) with the use of decongestant agents: a European pharmacovigilance analysis
Source: Pharmacol Rep. 2026 Jul 10;78(4):1264–74. doi: 10.1007/s43440-026-00877-z (PMC13437603; doi:10.1007/s43440-026-00877-z)
Supplement: Supplementary file 1 [file 43440_2026_877_MOESM1_ESM.docx]

**Supplementary Table 1.** List of decongestants by ATC code searched in the EudraVigilance database for events of RCVS or PRES. Source available at <https://atcddd.fhi.no/atc_ddd_index/>. ATC: Anatomical Therapeutic Chemical, PRES: posterior reversible encephalopathy syndrome, RCVS: reversible cerebral vasoconstriction syndrome.

| **ATC code** | **Name** |
| --- | --- |
| R01AA02 | [Cyclopentamine](https://atcddd.fhi.no/atc_ddd_index/?code=R01AA02&showdescription=yes) |
| R01AA03 | [Ephedrine](https://atcddd.fhi.no/atc_ddd_index/?code=R01AA03&showdescription=yes) |
| R01AA04 | [Phenylephrine](https://atcddd.fhi.no/atc_ddd_index/?code=R01AA04&showdescription=yes) |
| R01AA05 | [Oxymetazoline](https://atcddd.fhi.no/atc_ddd_index/?code=R01AA05&showdescription=yes) |
| R01AA06 | [Tetryzoline](https://atcddd.fhi.no/atc_ddd_index/?code=R01AA06&showdescription=yes) |
| R01AA07 | [Xylometazoline](https://atcddd.fhi.no/atc_ddd_index/?code=R01AA07&showdescription=yes) |
| R01AA08 | [Naphazoline](https://atcddd.fhi.no/atc_ddd_index/?code=R01AA08&showdescription=yes) |
| R01AA09 | [Tramazoline](https://atcddd.fhi.no/atc_ddd_index/?code=R01AA09&showdescription=yes) |
| R01AA10 | [Metizoline](https://atcddd.fhi.no/atc_ddd_index/?code=R01AA10&showdescription=yes) |
| R01AA11 | [Tuaminoheptane](https://atcddd.fhi.no/atc_ddd_index/?code=R01AA11&showdescription=yes) |
| R01AA12 | [Fenoxazoline](https://atcddd.fhi.no/atc_ddd_index/?code=R01AA12&showdescription=yes) |
| R01AA13 | [Tymazoline](https://atcddd.fhi.no/atc_ddd_index/?code=R01AA13&showdescription=yes) |
| R01AA14 | [Epinephrine](https://atcddd.fhi.no/atc_ddd_index/?code=R01AA14&showdescription=yes) |
| R01AA15 | [Indanazoline](https://atcddd.fhi.no/atc_ddd_index/?code=R01AA15&showdescription=yes) |
| R01BA01 | [Phenylpropanolamine](https://atcddd.fhi.no/atc_ddd_index/?code=R01BA01&showdescription=yes) |
| R01BA02 | [Pseudoephedrine](https://atcddd.fhi.no/atc_ddd_index/?code=R01BA02&showdescription=yes) |
| R01BA03 | [Phenylephrine](https://atcddd.fhi.no/atc_ddd_index/?code=R01BA03&showdescription=yes) |
| R01BA51 | [Phenylpropanolamine, Combinations](https://atcddd.fhi.no/atc_ddd_index/?code=R01BA51&showdescription=yes) |
| R01BA52 | [Pseudoephedrine, Combinations](https://atcddd.fhi.no/atc_ddd_index/?code=R01BA52&showdescription=yes) |
| R01BA53 | [Phenylephrine, Combinations](https://atcddd.fhi.no/atc_ddd_index/?code=R01BA53&showdescription=yes) |

**Supplementary Table 2**. All adverse events reported among cases of RCVS and PRES distributed for the suspected decongestant and retrieved from EudraVigilance from 2001 to December 31^st^, 2025. All values were presented as numbers (N) and percentages (%)

EPHE: ephedrine, IBU: ibuprofen, ICSRs: individual case safety reports, OXY: oxymetazoline, PARA: paracetamol, PHEN: phenylephrine, PRES: posterior reversible encephalopathy syndrome, PSEUDO; pseudoephedrine, RCVS: reversible cerebral vasoconstriction syndrome, TRIP: triprolidine, XYLO: xylometazoline.

| ADVERSE EVENT | EPHE (N=27) | OXY (N=21) | PHEN (N=72) | PHEN and EPHE* (N=12) | PHEN and PSEUDO* (N=16) | PSE-IBU** (N=12) | PSE-PARA-TRIP** (N=5) | PSEUDO (N=37) | XYLO (N=76) | XYLO, PHEN, and PSEUDO* (N=6) | XYLO and PSEUDO* (N=2) | OVERALL (N=286) |
| --- | --- | --- | --- | --- | --- | --- | --- | --- | --- | --- | --- | --- |
| Agraphia | 1 (3.7%) | 0 (0%) | 0 (0%) | 0 (0%) | 0 (0%) | 0 (0%) | 0 (0%) | 0 (0%) | 0 (0%) | 0 (0%) | 0 (0%) | 1 (0.3%) |
| Blood pressure increased | 2 (7.4%) | 0 (0%) | 0 (0%) | 0 (0%) | 0 (0%) | 0 (0%) | 0 (0%) | 0 (0%) | 0 (0%) | 0 (0%) | 0 (0%) | 2 (0.7%) |
| Cerebral haematoma | 1 (3.7%) | 0 (0%) | 0 (0%) | 0 (0%) | 0 (0%) | 0 (0%) | 0 (0%) | 0 (0%) | 0 (0%) | 0 (0%) | 0 (0%) | 1 (0.3%) |
| Cerebral infarction | 1 (3.7%) | 0 (0%) | 0 (0%) | 0 (0%) | 0 (0%) | 0 (0%) | 0 (0%) | 1 (2.7%) | 1 (1.3%) | 0 (0%) | 0 (0%) | 3 (1.0%) |
| Cerebral vasoconstriction | 2 (7.4%) | 1 (4.8%) | 0 (0%) | 0 (0%) | 0 (0%) | 0 (0%) | 0 (0%) | 0 (0%) | 3 (3.9%) | 0 (0%) | 0 (0%) | 6 (2.1%) |
| Dysgraphia | 1 (3.7%) | 0 (0%) | 0 (0%) | 0 (0%) | 0 (0%) | 0 (0%) | 0 (0%) | 0 (0%) | 0 (0%) | 0 (0%) | 0 (0%) | 1 (0.3%) |
| Headache | 3 (11.1%) | 1 (4.8%) | 4 (5.6%) | 1 (8.3%) | 0 (0%) | 1 (8.3%) | 1 (20.0%) | 4 (10.8%) | 2 (2.6%) | 0 (0%) | 0 (0%) | 17 (5.9%) |
| Hypertension | 1 (3.7%) | 0 (0%) | 2 (2.8%) | 0 (0%) | 0 (0%) | 0 (0%) | 0 (0%) | 1 (2.7%) | 4 (5.3%) | 0 (0%) | 0 (0%) | 8 (2.8%) |
| Muscular weakness | 1 (3.7%) | 1 (4.8%) | 0 (0%) | 0 (0%) | 0 (0%) | 0 (0%) | 0 (0%) | 0 (0%) | 0 (0%) | 0 (0%) | 0 (0%) | 2 (0.7%) |
| Overdose | 1 (3.7%) | 0 (0%) | 0 (0%) | 0 (0%) | 0 (0%) | 0 (0%) | 0 (0%) | 0 (0%) | 0 (0%) | 0 (0%) | 0 (0%) | 1 (0.3%) |
| Paraplegia | 1 (3.7%) | 0 (0%) | 0 (0%) | 0 (0%) | 0 (0%) | 0 (0%) | 0 (0%) | 0 (0%) | 0 (0%) | 0 (0%) | 0 (0%) | 1 (0.3%) |
| Posterior reversible encephalopathy syndrome | 3 (11.1%) | 1 (4.8%) | 7 (9.7%) | 0 (0%) | 0 (0%) | 1 (8.3%) | 0 (0%) | 2 (5.4%) | 0 (0%) | 0 (0%) | 0 (0%) | 14 (4.9%) |
| Reversible cerebral vasoconstriction syndrome | 3 (11.1%) | 4 (19.0%) | 11 (15.3%) | 1 (8.3%) | 1 (6.3%) | 2 (16.7%) | 2 (40.0%) | 8 (21.6%) | 15 (19.7%) | 4 (66.7%) | 1 (50.0%) | 52 (18.2%) |
| Status epilepticus | 2 (7.4%) | 0 (0%) | 0 (0%) | 0 (0%) | 0 (0%) | 0 (0%) | 0 (0%) | 0 (0%) | 0 (0%) | 0 (0%) | 0 (0%) | 2 (0.7%) |
| Subarachnoid haemorrhage | 1 (3.7%) | 1 (4.8%) | 0 (0%) | 1 (8.3%) | 1 (6.3%) | 0 (0%) | 0 (0%) | 1 (2.7%) | 5 (6.6%) | 1 (16.7%) | 1 (50.0%) | 12 (4.2%) |
| Vision blurred | 2 (7.4%) | 0 (0%) | 0 (0%) | 0 (0%) | 0 (0%) | 0 (0%) | 0 (0%) | 0 (0%) | 0 (0%) | 0 (0%) | 0 (0%) | 2 (0.7%) |
| Vomiting | 1 (3.7%) | 1 (4.8%) | 1 (1.4%) | 0 (0%) | 0 (0%) | 0 (0%) | 0 (0%) | 2 (5.4%) | 0 (0%) | 0 (0%) | 0 (0%) | 5 (1.7%) |
| Anxiety | 0 (0%) | 1 (4.8%) | 0 (0%) | 0 (0%) | 0 (0%) | 0 (0%) | 0 (0%) | 0 (0%) | 0 (0%) | 0 (0%) | 0 (0%) | 1 (0.3%) |
| Drug interaction | 0 (0%) | 2 (9.5%) | 0 (0%) | 0 (0%) | 0 (0%) | 0 (0%) | 0 (0%) | 0 (0%) | 2 (2.6%) | 0 (0%) | 0 (0%) | 4 (1.4%) |
| Hyperacusis | 0 (0%) | 1 (4.8%) | 0 (0%) | 0 (0%) | 0 (0%) | 0 (0%) | 0 (0%) | 0 (0%) | 0 (0%) | 0 (0%) | 0 (0%) | 1 (0.3%) |
| Magnetic resonance imaging abnormal | 0 (0%) | 1 (4.8%) | 0 (0%) | 0 (0%) | 0 (0%) | 0 (0%) | 0 (0%) | 0 (0%) | 0 (0%) | 0 (0%) | 0 (0%) | 1 (0.3%) |
| Nausea | 0 (0%) | 1 (4.8%) | 0 (0%) | 1 (8.3%) | 0 (0%) | 0 (0%) | 0 (0%) | 2 (5.4%) | 0 (0%) | 0 (0%) | 0 (0%) | 4 (1.4%) |
| Phobia | 0 (0%) | 1 (4.8%) | 0 (0%) | 0 (0%) | 0 (0%) | 0 (0%) | 0 (0%) | 0 (0%) | 0 (0%) | 0 (0%) | 0 (0%) | 1 (0.3%) |
| Photophobia | 0 (0%) | 1 (4.8%) | 0 (0%) | 0 (0%) | 0 (0%) | 0 (0%) | 0 (0%) | 0 (0%) | 0 (0%) | 0 (0%) | 0 (0%) | 1 (0.3%) |
| Seizure | 0 (0%) | 1 (4.8%) | 0 (0%) | 0 (0%) | 0 (0%) | 0 (0%) | 0 (0%) | 0 (0%) | 0 (0%) | 0 (0%) | 0 (0%) | 1 (0.3%) |
| Thunderclap headache | 0 (0%) | 2 (9.5%) | 1 (1.4%) | 0 (0%) | 1 (6.3%) | 0 (0%) | 0 (0%) | 1 (2.7%) | 6 (7.9%) | 0 (0%) | 0 (0%) | 11 (3.8%) |
| Altered state of consciousness | 0 (0%) | 0 (0%) | 1 (1.4%) | 0 (0%) | 0 (0%) | 0 (0%) | 0 (0%) | 0 (0%) | 0 (0%) | 0 (0%) | 0 (0%) | 1 (0.3%) |
| Amnesia | 0 (0%) | 0 (0%) | 1 (1.4%) | 0 (0%) | 0 (0%) | 0 (0%) | 0 (0%) | 0 (0%) | 0 (0%) | 0 (0%) | 0 (0%) | 1 (0.3%) |
| Anal incontinence | 0 (0%) | 0 (0%) | 1 (1.4%) | 0 (0%) | 1 (6.3%) | 0 (0%) | 0 (0%) | 0 (0%) | 0 (0%) | 0 (0%) | 0 (0%) | 2 (0.7%) |
| Aphasia | 0 (0%) | 0 (0%) | 2 (2.8%) | 0 (0%) | 1 (6.3%) | 0 (0%) | 0 (0%) | 1 (2.7%) | 0 (0%) | 0 (0%) | 0 (0%) | 4 (1.4%) |
| Asthenia | 0 (0%) | 0 (0%) | 1 (1.4%) | 0 (0%) | 0 (0%) | 0 (0%) | 0 (0%) | 0 (0%) | 0 (0%) | 0 (0%) | 0 (0%) | 1 (0.3%) |
| Ataxia | 0 (0%) | 0 (0%) | 1 (1.4%) | 0 (0%) | 1 (6.3%) | 0 (0%) | 0 (0%) | 0 (0%) | 0 (0%) | 0 (0%) | 0 (0%) | 2 (0.7%) |
| Blood pressure systolic increased | 0 (0%) | 0 (0%) | 1 (1.4%) | 0 (0%) | 1 (6.3%) | 0 (0%) | 0 (0%) | 0 (0%) | 0 (0%) | 0 (0%) | 0 (0%) | 2 (0.7%) |
| Bradycardia | 0 (0%) | 0 (0%) | 1 (1.4%) | 0 (0%) | 0 (0%) | 0 (0%) | 0 (0%) | 0 (0%) | 0 (0%) | 0 (0%) | 0 (0%) | 1 (0.3%) |
| Cardiac failure | 0 (0%) | 0 (0%) | 1 (1.4%) | 0 (0%) | 0 (0%) | 0 (0%) | 0 (0%) | 0 (0%) | 0 (0%) | 0 (0%) | 0 (0%) | 1 (0.3%) |
| Cerebral haemorrhage | 0 (0%) | 0 (0%) | 7 (9.7%) | 0 (0%) | 1 (6.3%) | 0 (0%) | 0 (0%) | 0 (0%) | 0 (0%) | 0 (0%) | 0 (0%) | 8 (2.8%) |
| Cerebrovascular accident | 0 (0%) | 0 (0%) | 1 (1.4%) | 1 (8.3%) | 1 (6.3%) | 0 (0%) | 1 (20.0%) | 0 (0%) | 0 (0%) | 0 (0%) | 0 (0%) | 4 (1.4%) |
| Confusional state | 0 (0%) | 0 (0%) | 1 (1.4%) | 0 (0%) | 0 (0%) | 0 (0%) | 0 (0%) | 1 (2.7%) | 0 (0%) | 0 (0%) | 0 (0%) | 2 (0.7%) |
| Epilepsy | 0 (0%) | 0 (0%) | 1 (1.4%) | 0 (0%) | 0 (0%) | 0 (0%) | 0 (0%) | 1 (2.7%) | 0 (0%) | 0 (0%) | 0 (0%) | 2 (0.7%) |
| Extensor plantar response | 0 (0%) | 0 (0%) | 1 (1.4%) | 0 (0%) | 0 (0%) | 0 (0%) | 0 (0%) | 0 (0%) | 0 (0%) | 0 (0%) | 0 (0%) | 1 (0.3%) |
| Eye disorder | 0 (0%) | 0 (0%) | 1 (1.4%) | 0 (0%) | 1 (6.3%) | 0 (0%) | 0 (0%) | 0 (0%) | 0 (0%) | 0 (0%) | 0 (0%) | 2 (0.7%) |
| Gaze palsy | 0 (0%) | 0 (0%) | 1 (1.4%) | 0 (0%) | 1 (6.3%) | 0 (0%) | 0 (0%) | 0 (0%) | 0 (0%) | 0 (0%) | 0 (0%) | 2 (0.7%) |
| Haematoma | 0 (0%) | 0 (0%) | 1 (1.4%) | 0 (0%) | 0 (0%) | 0 (0%) | 0 (0%) | 0 (0%) | 0 (0%) | 0 (0%) | 0 (0%) | 1 (0.3%) |
| Hemiparesis | 0 (0%) | 0 (0%) | 3 (4.2%) | 0 (0%) | 1 (6.3%) | 0 (0%) | 0 (0%) | 0 (0%) | 0 (0%) | 0 (0%) | 0 (0%) | 4 (1.4%) |
| Hemiplegia | 0 (0%) | 0 (0%) | 1 (1.4%) | 0 (0%) | 0 (0%) | 0 (0%) | 0 (0%) | 0 (0%) | 0 (0%) | 0 (0%) | 0 (0%) | 1 (0.3%) |
| Hyperreflexia | 0 (0%) | 0 (0%) | 1 (1.4%) | 0 (0%) | 0 (0%) | 0 (0%) | 0 (0%) | 0 (0%) | 0 (0%) | 0 (0%) | 0 (0%) | 1 (0.3%) |
| Hypertensive emergency | 0 (0%) | 0 (0%) | 1 (1.4%) | 0 (0%) | 0 (0%) | 0 (0%) | 0 (0%) | 0 (0%) | 0 (0%) | 0 (0%) | 0 (0%) | 1 (0.3%) |
| Hypertonia | 0 (0%) | 0 (0%) | 1 (1.4%) | 0 (0%) | 0 (0%) | 0 (0%) | 0 (0%) | 0 (0%) | 0 (0%) | 0 (0%) | 0 (0%) | 1 (0.3%) |
| Inappropriate affect | 0 (0%) | 0 (0%) | 3 (4.2%) | 0 (0%) | 0 (0%) | 0 (0%) | 0 (0%) | 0 (0%) | 0 (0%) | 0 (0%) | 0 (0%) | 3 (1.0%) |
| Intentional product misuse | 0 (0%) | 0 (0%) | 2 (2.8%) | 0 (0%) | 0 (0%) | 0 (0%) | 0 (0%) | 0 (0%) | 1 (1.3%) | 0 (0%) | 0 (0%) | 3 (1.0%) |
| Maternal exposure before pregnancy | 0 (0%) | 0 (0%) | 1 (1.4%) | 0 (0%) | 0 (0%) | 0 (0%) | 0 (0%) | 0 (0%) | 0 (0%) | 0 (0%) | 0 (0%) | 1 (0.3%) |
| Neurological decompensation | 0 (0%) | 0 (0%) | 1 (1.4%) | 0 (0%) | 0 (0%) | 0 (0%) | 0 (0%) | 0 (0%) | 0 (0%) | 0 (0%) | 0 (0%) | 1 (0.3%) |
| Off label use | 0 (0%) | 0 (0%) | 1 (1.4%) | 0 (0%) | 0 (0%) | 0 (0%) | 0 (0%) | 0 (0%) | 0 (0%) | 0 (0%) | 0 (0%) | 1 (0.3%) |
| Oscillopsia | 0 (0%) | 0 (0%) | 1 (1.4%) | 0 (0%) | 0 (0%) | 0 (0%) | 0 (0%) | 0 (0%) | 0 (0%) | 0 (0%) | 0 (0%) | 1 (0.3%) |
| Papilloedema | 0 (0%) | 0 (0%) | 1 (1.4%) | 0 (0%) | 0 (0%) | 0 (0%) | 0 (0%) | 0 (0%) | 0 (0%) | 0 (0%) | 0 (0%) | 1 (0.3%) |
| Sinus tachycardia | 0 (0%) | 0 (0%) | 1 (1.4%) | 0 (0%) | 0 (0%) | 0 (0%) | 0 (0%) | 0 (0%) | 0 (0%) | 0 (0%) | 0 (0%) | 1 (0.3%) |
| Unresponsive to stimuli | 0 (0%) | 0 (0%) | 1 (1.4%) | 0 (0%) | 0 (0%) | 0 (0%) | 0 (0%) | 0 (0%) | 0 (0%) | 0 (0%) | 0 (0%) | 1 (0.3%) |
| Vasogenic cerebral oedema | 0 (0%) | 0 (0%) | 1 (1.4%) | 0 (0%) | 0 (0%) | 0 (0%) | 0 (0%) | 0 (0%) | 0 (0%) | 0 (0%) | 0 (0%) | 1 (0.3%) |
| Visual impairment | 0 (0%) | 0 (0%) | 2 (2.8%) | 0 (0%) | 0 (0%) | 0 (0%) | 0 (0%) | 0 (0%) | 0 (0%) | 0 (0%) | 0 (0%) | 2 (0.7%) |
| Arterial stenosis | 0 (0%) | 0 (0%) | 0 (0%) | 1 (8.3%) | 0 (0%) | 0 (0%) | 0 (0%) | 0 (0%) | 0 (0%) | 0 (0%) | 0 (0%) | 1 (0.3%) |
| Blood prolactin increased | 0 (0%) | 0 (0%) | 0 (0%) | 1 (8.3%) | 0 (0%) | 0 (0%) | 0 (0%) | 0 (0%) | 0 (0%) | 0 (0%) | 0 (0%) | 1 (0.3%) |
| Diabetes insipidus | 0 (0%) | 0 (0%) | 0 (0%) | 1 (8.3%) | 0 (0%) | 0 (0%) | 0 (0%) | 0 (0%) | 0 (0%) | 0 (0%) | 0 (0%) | 1 (0.3%) |
| Pituitary apoplexy | 0 (0%) | 0 (0%) | 0 (0%) | 1 (8.3%) | 0 (0%) | 0 (0%) | 0 (0%) | 0 (0%) | 0 (0%) | 0 (0%) | 0 (0%) | 1 (0.3%) |
| Pituitary enlargement | 0 (0%) | 0 (0%) | 0 (0%) | 1 (8.3%) | 0 (0%) | 0 (0%) | 0 (0%) | 0 (0%) | 0 (0%) | 0 (0%) | 0 (0%) | 1 (0.3%) |
| Polyuria | 0 (0%) | 0 (0%) | 0 (0%) | 1 (8.3%) | 0 (0%) | 0 (0%) | 0 (0%) | 0 (0%) | 0 (0%) | 0 (0%) | 0 (0%) | 1 (0.3%) |
| Urine osmolarity decreased | 0 (0%) | 0 (0%) | 0 (0%) | 1 (8.3%) | 0 (0%) | 0 (0%) | 0 (0%) | 0 (0%) | 0 (0%) | 0 (0%) | 0 (0%) | 1 (0.3%) |
| Angiogram cerebral abnormal | 0 (0%) | 0 (0%) | 0 (0%) | 0 (0%) | 1 (6.3%) | 0 (0%) | 0 (0%) | 1 (2.7%) | 0 (0%) | 0 (0%) | 0 (0%) | 2 (0.7%) |
| Blood potassium decreased | 0 (0%) | 0 (0%) | 0 (0%) | 0 (0%) | 1 (6.3%) | 0 (0%) | 0 (0%) | 0 (0%) | 0 (0%) | 0 (0%) | 0 (0%) | 1 (0.3%) |
| Intraventricular haemorrhage | 0 (0%) | 0 (0%) | 0 (0%) | 0 (0%) | 1 (6.3%) | 0 (0%) | 0 (0%) | 0 (0%) | 0 (0%) | 0 (0%) | 0 (0%) | 1 (0.3%) |
| Urinary incontinence | 0 (0%) | 0 (0%) | 0 (0%) | 0 (0%) | 1 (6.3%) | 0 (0%) | 0 (0%) | 0 (0%) | 0 (0%) | 0 (0%) | 0 (0%) | 1 (0.3%) |
| Ageusia | 0 (0%) | 0 (0%) | 0 (0%) | 0 (0%) | 0 (0%) | 1 (8.3%) | 0 (0%) | 0 (0%) | 0 (0%) | 0 (0%) | 0 (0%) | 1 (0.3%) |
| Anosmia | 0 (0%) | 0 (0%) | 0 (0%) | 0 (0%) | 0 (0%) | 1 (8.3%) | 0 (0%) | 0 (0%) | 0 (0%) | 0 (0%) | 0 (0%) | 1 (0.3%) |
| Dizziness | 0 (0%) | 0 (0%) | 0 (0%) | 0 (0%) | 0 (0%) | 1 (8.3%) | 0 (0%) | 0 (0%) | 0 (0%) | 0 (0%) | 0 (0%) | 1 (0.3%) |
| Encephalitis haemorrhagic | 0 (0%) | 0 (0%) | 0 (0%) | 0 (0%) | 0 (0%) | 1 (8.3%) | 0 (0%) | 0 (0%) | 0 (0%) | 0 (0%) | 0 (0%) | 1 (0.3%) |
| Hypertensive crisis | 0 (0%) | 0 (0%) | 0 (0%) | 0 (0%) | 0 (0%) | 1 (8.3%) | 0 (0%) | 0 (0%) | 0 (0%) | 0 (0%) | 0 (0%) | 1 (0.3%) |
| Meningism | 0 (0%) | 0 (0%) | 0 (0%) | 0 (0%) | 0 (0%) | 1 (8.3%) | 0 (0%) | 0 (0%) | 0 (0%) | 0 (0%) | 0 (0%) | 1 (0.3%) |
| Paraesthesia | 0 (0%) | 0 (0%) | 0 (0%) | 0 (0%) | 0 (0%) | 1 (8.3%) | 0 (0%) | 0 (0%) | 0 (0%) | 0 (0%) | 0 (0%) | 1 (0.3%) |
| Subdural haematoma | 0 (0%) | 0 (0%) | 0 (0%) | 0 (0%) | 0 (0%) | 1 (8.3%) | 0 (0%) | 0 (0%) | 0 (0%) | 0 (0%) | 0 (0%) | 1 (0.3%) |
| Drug dependence | 0 (0%) | 0 (0%) | 0 (0%) | 0 (0%) | 0 (0%) | 0 (0%) | 1 (20.0%) | 0 (0%) | 0 (0%) | 0 (0%) | 0 (0%) | 1 (0.3%) |
| Cerebral vasodilatation | 0 (0%) | 0 (0%) | 0 (0%) | 0 (0%) | 0 (0%) | 0 (0%) | 0 (0%) | 1 (2.7%) | 0 (0%) | 0 (0%) | 0 (0%) | 1 (0.3%) |
| Cerebrovascular stenosis | 0 (0%) | 0 (0%) | 0 (0%) | 0 (0%) | 0 (0%) | 0 (0%) | 0 (0%) | 1 (2.7%) | 0 (0%) | 0 (0%) | 0 (0%) | 1 (0.3%) |
| Disorientation | 0 (0%) | 0 (0%) | 0 (0%) | 0 (0%) | 0 (0%) | 0 (0%) | 0 (0%) | 1 (2.7%) | 0 (0%) | 0 (0%) | 0 (0%) | 1 (0.3%) |
| Humerus fracture | 0 (0%) | 0 (0%) | 0 (0%) | 0 (0%) | 0 (0%) | 0 (0%) | 0 (0%) | 1 (2.7%) | 0 (0%) | 0 (0%) | 0 (0%) | 1 (0.3%) |
| Leukocytosis | 0 (0%) | 0 (0%) | 0 (0%) | 0 (0%) | 0 (0%) | 0 (0%) | 0 (0%) | 1 (2.7%) | 0 (0%) | 0 (0%) | 0 (0%) | 1 (0.3%) |
| Migraine | 0 (0%) | 0 (0%) | 0 (0%) | 0 (0%) | 0 (0%) | 0 (0%) | 0 (0%) | 1 (2.7%) | 0 (0%) | 0 (0%) | 0 (0%) | 1 (0.3%) |
| Normocytic anaemia | 0 (0%) | 0 (0%) | 0 (0%) | 0 (0%) | 0 (0%) | 0 (0%) | 0 (0%) | 1 (2.7%) | 0 (0%) | 0 (0%) | 0 (0%) | 1 (0.3%) |
| Pain | 0 (0%) | 0 (0%) | 0 (0%) | 0 (0%) | 0 (0%) | 0 (0%) | 0 (0%) | 1 (2.7%) | 0 (0%) | 0 (0%) | 0 (0%) | 1 (0.3%) |
| Paresis | 0 (0%) | 0 (0%) | 0 (0%) | 0 (0%) | 0 (0%) | 0 (0%) | 0 (0%) | 1 (2.7%) | 0 (0%) | 0 (0%) | 0 (0%) | 1 (0.3%) |
| Rhinorrhoea | 0 (0%) | 0 (0%) | 0 (0%) | 0 (0%) | 0 (0%) | 0 (0%) | 0 (0%) | 1 (2.7%) | 6 (7.9%) | 0 (0%) | 0 (0%) | 7 (2.4%) |
| Transient global amnesia | 0 (0%) | 0 (0%) | 0 (0%) | 0 (0%) | 0 (0%) | 0 (0%) | 0 (0%) | 1 (2.7%) | 0 (0%) | 0 (0%) | 0 (0%) | 1 (0.3%) |
| Allergy to animal | 0 (0%) | 0 (0%) | 0 (0%) | 0 (0%) | 0 (0%) | 0 (0%) | 0 (0%) | 0 (0%) | 3 (3.9%) | 0 (0%) | 0 (0%) | 3 (1.0%) |
| Cerebral artery stenosis | 0 (0%) | 0 (0%) | 0 (0%) | 0 (0%) | 0 (0%) | 0 (0%) | 0 (0%) | 0 (0%) | 4 (5.3%) | 0 (0%) | 0 (0%) | 4 (1.4%) |
| Horner's syndrome | 0 (0%) | 0 (0%) | 0 (0%) | 0 (0%) | 0 (0%) | 0 (0%) | 0 (0%) | 0 (0%) | 1 (1.3%) | 0 (0%) | 0 (0%) | 1 (0.3%) |
| Ischaemic stroke | 0 (0%) | 0 (0%) | 0 (0%) | 0 (0%) | 0 (0%) | 0 (0%) | 0 (0%) | 0 (0%) | 1 (1.3%) | 0 (0%) | 0 (0%) | 1 (0.3%) |
| Nasal mucosal disorder | 0 (0%) | 0 (0%) | 0 (0%) | 0 (0%) | 0 (0%) | 0 (0%) | 0 (0%) | 0 (0%) | 4 (5.3%) | 0 (0%) | 0 (0%) | 4 (1.4%) |
| Nasal septum deviation | 0 (0%) | 0 (0%) | 0 (0%) | 0 (0%) | 0 (0%) | 0 (0%) | 0 (0%) | 0 (0%) | 4 (5.3%) | 0 (0%) | 0 (0%) | 4 (1.4%) |
| Nasal turbinate hypertrophy | 0 (0%) | 0 (0%) | 0 (0%) | 0 (0%) | 0 (0%) | 0 (0%) | 0 (0%) | 0 (0%) | 4 (5.3%) | 0 (0%) | 0 (0%) | 4 (1.4%) |
| Product use in unapproved indication | 0 (0%) | 0 (0%) | 0 (0%) | 0 (0%) | 0 (0%) | 0 (0%) | 0 (0%) | 0 (0%) | 4 (5.3%) | 0 (0%) | 0 (0%) | 4 (1.4%) |
| Rebound nasal congestion | 0 (0%) | 0 (0%) | 0 (0%) | 0 (0%) | 0 (0%) | 0 (0%) | 0 (0%) | 0 (0%) | 5 (6.6%) | 0 (0%) | 0 (0%) | 5 (1.7%) |
| Vertebrobasilar artery stenosis | 0 (0%) | 0 (0%) | 0 (0%) | 0 (0%) | 0 (0%) | 0 (0%) | 0 (0%) | 0 (0%) | 1 (1.3%) | 0 (0%) | 0 (0%) | 1 (0.3%) |
| Wrong technique in product usage process | 0 (0%) | 0 (0%) | 0 (0%) | 0 (0%) | 0 (0%) | 0 (0%) | 0 (0%) | 0 (0%) | 0 (0%) | 1 (16.7%) | 0 (0%) | 1 (0.3%) |

* ICSRs reporting different formulations containing a single decongestant (more than 1 decongestant reported as suspected drug).
**ICSRs reporting a single formulation containing more active ingredients.
